# Multi-omics profiling reveals a distinctive epigenome signature for high-risk acute promyelocytic leukemia

**DOI:** 10.18632/oncotarget.25429

**Published:** 2018-05-22

**Authors:** Abhishek A. Singh, Francesca Petraglia, Angela Nebbioso, Guoqiang Yi, Mariarosaria Conte, Sergio Valente, Amit Mandoli, Lucia Scisciola, Rik Lindeboom, Hinri Kerstens, Eva M. Janssen-Megens, Farzin Pourfarzad, Ehsan Habibi, Kim Berentsen, Bowon Kim, Colin Logie, Simon Heath, Albertus T.J. Wierenga, Laura Clarke, Paul Flicek, Joop H. Jansen, Taco Kuijpers, Marie Laure Yaspo, Veronique Della Valle, Olivier Bernard, Ivo Gut, Edo Vellenga, Hendrik G. Stunnenberg, Antonello Mai, Lucia Altucci, Joost H.A. Martens

**Affiliations:** ^1^ Department of Molecular Biology, Radboud University, Nijmegen, Netherlands; ^2^ Dipartimento di Biochimica Biofisica e Patologia Generale, Università degli Studi della Campania Luigi Vanvitelli, Napoli, Italy; ^3^ IRCCS SDN, Napoli Via E. Gianturco, Napoli, Italy; ^4^ Dipartimento di Chimica e Tecnologie del Farmaco ‘Sapienza’ Università, Roma, Italy; ^5^ Department of Blood Cell Research, Sanquin Research and Landsteiner Laboratory, Academic Medical Center, University of Amsterdam, Amsterdam, Netherlands; ^6^ Centro Nacional de Análisis Genómico, Barcelona, Spain; ^7^ Department of Hematology, University of Groningen and University Medical Center Groningen, Groningen, Netherlands; ^8^ European Molecular Biology Laboratory, European Bioinformatics Institute, Wellcome Trust Genome Campus, Hinxton, United Kingdom; ^9^ Department of Laboratory Medicine, Radboud UMC, Nijmegen, Netherlands; ^10^ Max Planck Institute for Molecular Genetics, Berlin, Germany; ^11^ INSERM U1170, Universtité Paris-Saclay, Institut Gustave Roussy, Equipe Labellisée Ligue Nationale Contre le Cancer (LNCC), Paris, France; ^12^ Pasteur Institute, Cenci-Bolognetti Foundation, Sapienza University of Rome, Roma, Italy

**Keywords:** epigenome, acute promyelocytic leukemia (APL), PML-RARA, high-risk APL, epi-drugs

## Abstract

Epigenomic alterations have been associated with both pathogenesis and progression of cancer. Here, we analyzed the epigenome of two high-risk APL (hrAPL) patients and compared it to non-high-risk APL cases. Despite the lack of common genetic signatures, we found that human hrAPL blasts from patients with extremely poor prognosis display specific patterns of histone H3 acetylation, specifically hyperacetylation at a common set of enhancer regions. In addition, unique profiles of the repressive marks H3K27me3 and DNA methylation were exposed in high-risk APLs. Epigenetic comparison with low/intermediate-risk APLs and AMLs revealed hrAPL-specific patterns of histone acetylation and DNA methylation, suggesting these could be further developed into markers for clinical identification. The epigenetic drug MC2884, a newly generated general HAT/EZH2 inhibitor, induces apoptosis of high-risk APL blasts and reshapes their epigenomes by targeting both active and repressive marks. Together, our analysis uncovers distinctive epigenome signatures of hrAPL patients, and provides proof of concept for use of epigenome profiling coupled to epigenetic drugs to ‘personalize’ precision medicine.

## INTRODUCTION

Acute promyelocytic leukemia (APL) is a subtype of acute myeloid leukemia (AML) and accounts for ∼10% of all AML cases. APL is most often characterized by the chromosomal translocation t(15;17) that fuses the *PML* gene on chromosome 15 to the retinoic acid receptor α (*RARA*) gene on chromosome 17 resulting in the transcription of the PML-RARA oncofusion protein [1, 2]. The PML-RARA fusion has been reported to be present in >95% of APL cases [1, 3]. In normal cells, PML regulates a wide range of proteins including transcription factors and ensures normal functioning of biological processes such as DNA damage response and microorganism resistance [4], while RARA-involved pathways are critical for terminal differentiation of myeloid cells [5]. The formation of the PML-RARA oncofusion protein disrupts normal functioning of both fusion partners expressed by wild-type alleles [6] and induces a maturation block at the promyelocytic stage [7].

The PML-RARA oncofusion protein has been proposed to recruit epigenetic modifiers, like histone deacetylase complexes such as SMRT [8] and N-CoR [9], DNA methyltransferases [10], and the repressive histone methyltransferases SUV39HI [11] and PRC2 [3], to provoke alterations in chromatin structure. Genome-wide analyses revealed that the majority of PML-RARA binding sites have low histone H3 acetylation [12–14], suggesting a main role for histone deacetylases in PML-RARA leukemogenesis.

Although the PML-RARA fusion event is a major driving force in APL, the treatment of APL patients is clinically different according to risk classification. In general, APL can be subdivided into three risk categories based on white blood cell (WBC) and platelet count. Low risk APL patients have a WBC count lower than 10,000/µL and a platelet count over 40,000/µL, intermediate-risk APL patients carry a WBC count under 10,000/µL but a platelet count lower than 40,000/µL, while high-risk APL patients have a WBC count >10,000/µL. Despite significant advances in the treatment of APL, the category of high-risk APL still poses pressing challenges.

Pharmacological doses of All-*trans* retinoic acid (ATRA) and/or arsenic trioxide (ATO) are widely used by clinicians in treating low- and intermediate-risk APL patients [15, 16]. ATO and ATRA act on PML and RARA, respectively, and promote degradation of the PML-RARA oncofusion protein. ATRA adopts the proteasome-mediated pathway [17] and caspases [18] to degrade PML-RARA, and ATO induces sumoylation of the PML moiety [19]. Depletion of the PML-RARA oncofusion protein leads to loss of recruitment of epigenetic repressors and remodels the repressive chromatin environment at PML-RARA binding sites, creating a more accessible chromatin state [13]. In contrast to low/intermediate-risk APL patients, high-risk APL patients generally show a higher relapse rate, and are currently often treated with cytotoxic chemotherapy in combination with ATRA/ATO [20].

By performing an in-depth transcriptomic and epigenomic analysis, we aimed to gain further insights into these high-risk APLs (hrAPLs) that might ultimately help in improving their prognosis. We used ChIP-seq to identify H3K4me1, H3K4me3, H3K27ac, H3K9/14ac, H3K27me3, and H3K36me3 enriched regions in patient blasts from two high-risk APLs, and also conducted whole-genome-bisulfite sequencing (WGBS) and RNA-seq to probe DNA methylation and gene expression profiles, respectively. We compared the epigenomes of high-risk APL samples with non-high-risk APL samples as well as with other progenitor cells from the normal myeloid compartment, revealing a hyperacetylation signature at a defined set of enhancer regions in hrAPLs. As we also uncovered hrAPL-specific alterations for H3K27me3, our analysis suggested that a combination of epigenetic drugs that affects acetyltransferase and H3K27 methyltransferase activity might be beneficial. Using the novel HAT/EZH2 inhibitor MC2884 confirmed that altering these hrAPL-specific epigenomic features could indeed significantly inhibit cell growth.

## RESULTS

### The genomic spectrum of two high-risk APLs

**Figure 1:**
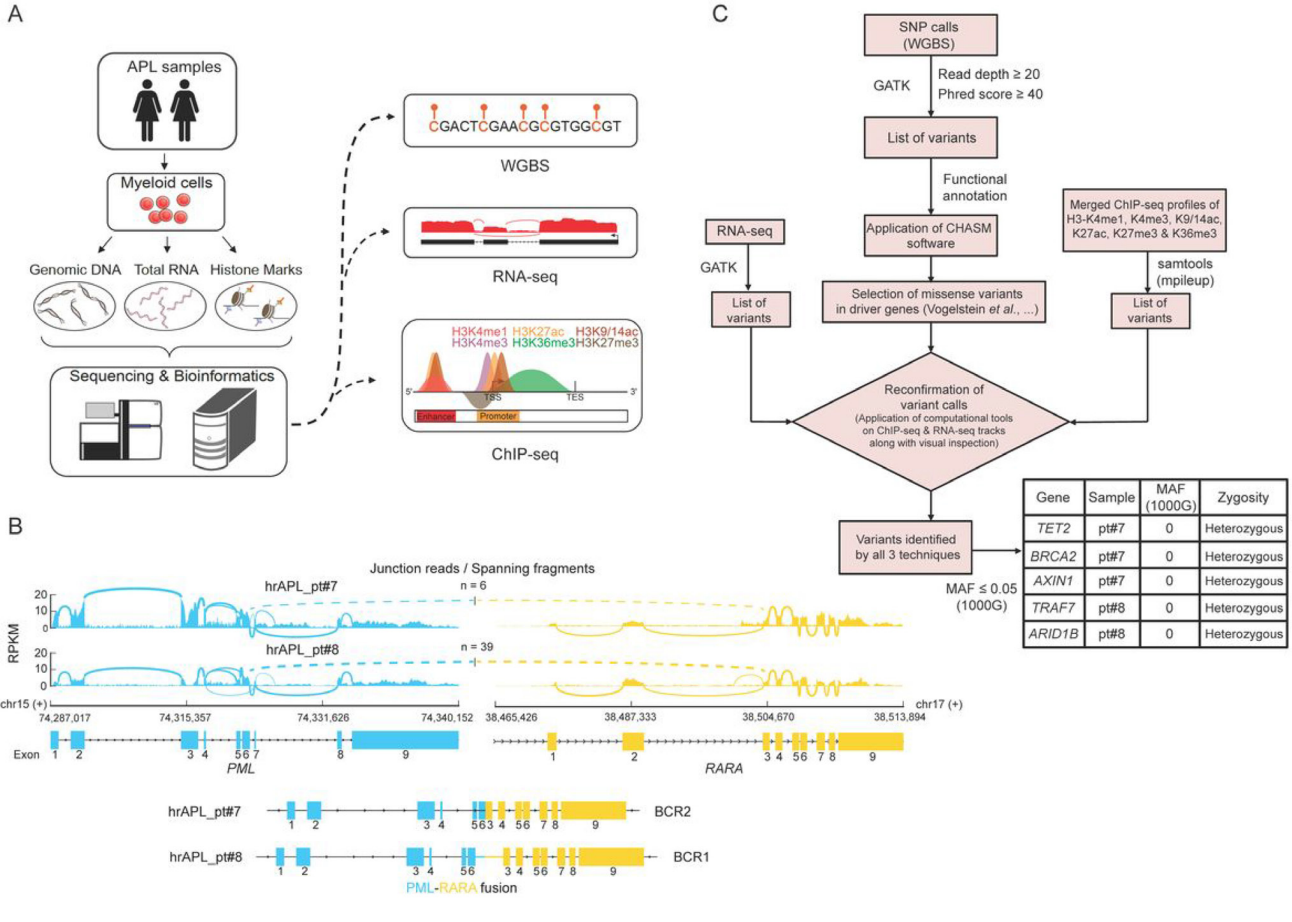
Genomic analysis of two high-risk APLs (hrAPLs) (**A**) Schematic depiction of the experimental setup and analysis workflow for two hrAPLs. (**B**) Overview of the PML-RARA isoforms expressed in patient #7 (pt#7) and #8 (pt#8). (**C**) Diagram of the analysis workflow to identify genomic variants in the two low OS APLs #7 and #8. The table depicts the top candidate genetic variants identified in pt#7 and pt#8.

To investigate the molecular determinants of high-risk APLs, we analyzed the genome and epigenome of two rare primary APL samples of patients that were resistant to standard ATRA plus chemo treatment and presented extremely high white blood cell counts (>10,000/µL) (Figure 1A). We confirmed transcription of the PML-RARA fusion in both samples, which revealed expression of the BCR2 isoform in patient 7 (pt#7) and BCR1 in patient 8 (pt#8) (Figure 1B). In addition, we used SNP calls inferred from WGBS data to look for common gene variants in the RARA moiety of the two hrAPLs, that could potentially explain the absence of an ATRA response [21–24]. We found one rare gene variant signature with minor allele frequency (MAF) <0.007 in our two hrAPL samples, but as this variant was synonymous it was not expected to have functional effects on the RARA moiety.

To examine the full mutational landscape of the two hrAPL patients, we used variant calls from WGBS as our primary calls and applied additional thresholds of read depth (DP) ≥20 and Phred score ≥40. To have more confidence in our variant calls, we confirmed these calls using RNA-seq and/or ChIP-seq profiles of these two samples. Next, we looked for only missense variants and evaluated them using the CHASM program (http://www.cravat.us/), on a cut-off of MAF ≤0.05, which resulted in the identification of a range of mutations. Specifically, the analysis identified several candidate driving mutations such as *TET2* and *BRCA2*, but none of them was recurrent in the two patients (Figure 1C).

### A hyperacetylation signature typifies PML-RARA binding sites in high-risk APLs

**Figure 2:**
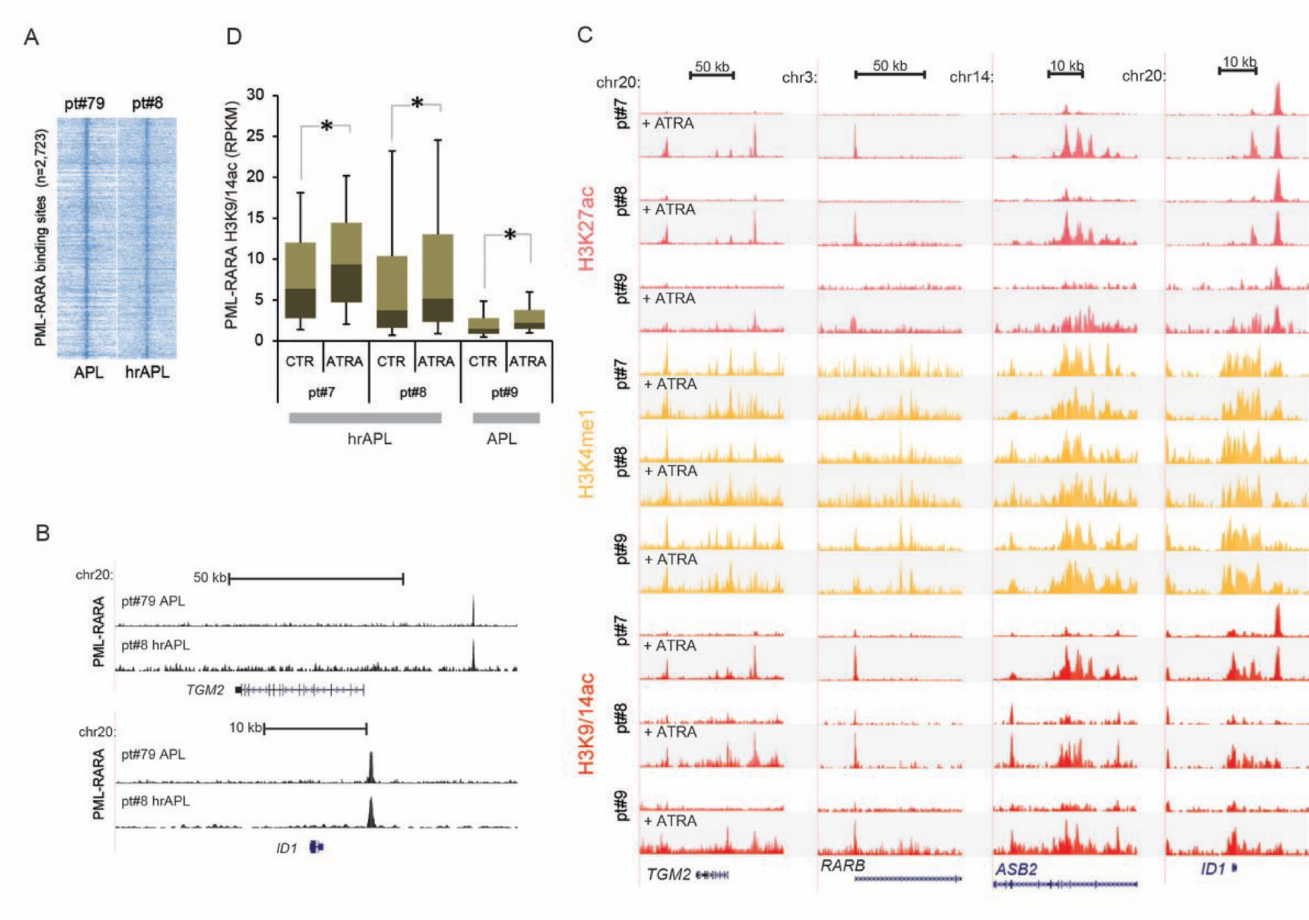
Epigenomic analysis of high-risk APLs (hrAPLs) (**A**) Heatmap of PML tag density at PML-RARA binding sites ± 10 kb, after PML ChIP-seq in one APL (pt#79) and one hrAPL (pt#8). (**B**) Genome browser screenshot of PML ChIP-seq results at the *TGM2* and *ID1* locus. (**C**) Overview of APL ChIP-seq (H3K27ac, H3K4me1, H3K9/14ac) data at the genomic regions of the *TGM2*, *RARB*, *ASB2* and *ID1* genes before and after *ex vivo* ATRA treatment. (**D**) Boxplot showing changes in H3K9/14ac before and after *ex vivo* treatment with ATRA at PML-RARA binding sites in one APL and two hrAPLs. ^*^ indicates significance at *p* < 0.01 using the Wilcoxon Signed-Rank test.

Given the absence of common secondary mutations, we focused on the epigenomic patterns observed in the two hrAPL patients and explored whether an ATRA response was still observed at the epigenetic level as compared to low/intermediate-risk APLs. The APL-associated oncofusion protein PML-RARA is able to recruit HDACs and establish a hypoacetylated chromatin signature, which can be alleviated by ATRA-induced PML-RARA degradation [13]. Hence, we first surveyed binding of PML-RARA in hrAPLs by ChIP-seq using an antibody against PML (the non-DNA-binding moiety of the PML-RARA fusion). Our analysis revealed occupancy of PML-RARA at previously identified high-confidence PML-RARA binding sites (*n* = 2,723) (Figure 2A) from the NB4 model cell line and low/intermediate-risk APLs [13], such as the *TGM2* and *ID1* genomic regions (Figure 2B). We generated additional epigenetic ChIP-seq profiles using *ex vivo* ATRA treated patient cells and examined epigenetic alterations at the same PML-RARA binding sites. This confirmed elevated histone H3 acetylation levels after PML-RARA depletion by ATRA treatment at the *TGM2, RARB, ASB2* and *ID1* loci (Figure 2C). Next, we further delved into global ATRA response at all 2,723 PML-RARA occupancy loci and found that generally H3K9/14ac levels increased in an ATRA-treatment-dependent manner in hrAPLs (pt#7 and pt#8) (Figure 2D). Interestingly, we noted significantly elevated steady-state levels of histone acetylation at PML-RARA binding sites in hrAPLs as compared to low/intermediate-risk APL (pt#9) (Figure 2D) in both control and ATRA-treated samples, suggesting that abnormal hyperacetylation at PML-RARA binding sites might be a crucial property of high-risk APLs.

### Hyperacetylation at enhancer and promoter regions in high-risk APLs

**Figure 3:**
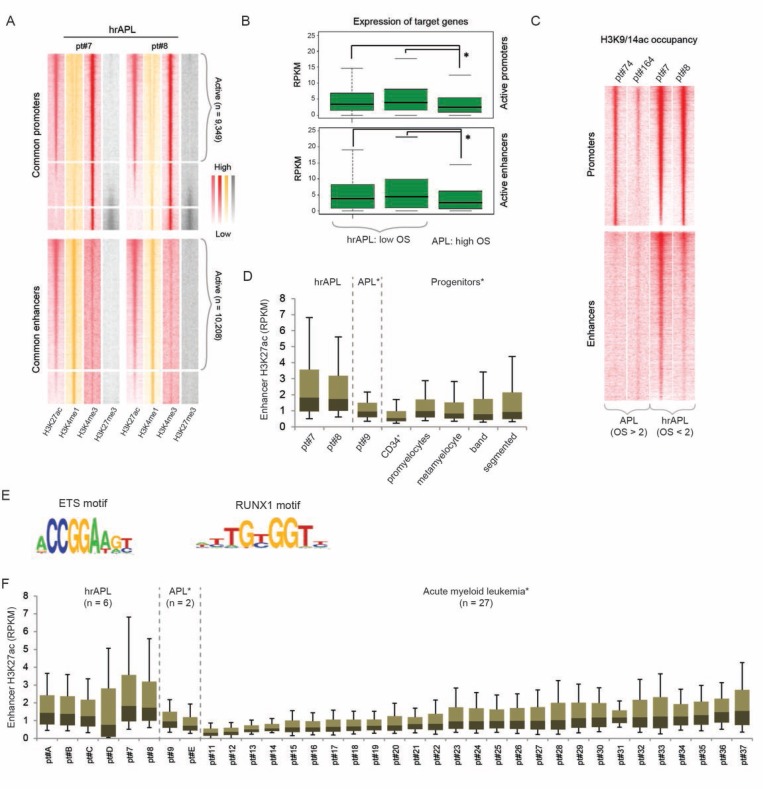
The histone acetylation signature in high-risk APLs (hrAPLs) (**A**) Heatmap of histone modification (H3K27ac, H3K4me1, H3K4me3 and H3K27me3) densities at promoter and enhancer regions defined in hrAPL #7 and #8. (**B**) Boxplot showing expression of genes associated with active promoter (top) and enhancer (bottom) regions defined in two hrAPLs (pt#7 and pt#8) and one APL (pt#9). (**C**) Heatmap of H3K9/14ac densities at active promoter and enhancer regions defined in two hrAPLs (pt#7 and pt#8) and two APLs (pt#74 and pt#164). (**D**) Boxplot showing H3K27ac levels of one primary APL (pt#9), 2 hrAPLs (pt#7 and pt#8), normal CD34^+^ cells and normal neutrophil progenitor populations (promyelocytes, metamyelocytes, band neutrophils and segmented neutrophils) at enhancer regions defined in hrAPLs. ^*^p < 0.01 for all pairwise comparisons between hrAPLs and other APLs/progenitors. (**E**) Motifs enriched at enhancer regions identified in hrAPLs as compared to random regions of equal size with similar nucleotide composition and a random set of ENCODE defined enhancers. (**F**) Boxplot showing H3K27ac levels of primary APLs, hrAPLs and 27 AMLs with a different genetic status at enhancer regions defined in hrAPLs. ^*^p < 0.01 for all pairwise comparisons between hrAPLs and other APLs/AMLs, excluding comparisons with AML pt#37.

To analyze whether increased acetylation (H3K9/14ac and H3K27ac) was a genome-wide feature, we identified all active hrAPL promoter and enhancer regions based on levels of the promoter mark H3K4me3 or the enhancer mark H3K4me1 in association with H3K27ac (Figure 3A). A total of 9,349 common active promoters (high H3K4me3 and H3K27ac, low H3K4me1 and H3K27me3) and 10,208 common active enhancers (high H3K4me1 and H3K27ac, low H3K4me3 and H3K27me3) were detected. In line with the positive correlation between histone H3 acetylation levels and gene expression, we observed that associated genes of active promoters and enhancers are expressed, but also that these target genes seem higher expressed in hrAPLs as compared to APL (Figure 3B). To further explore whether elevated acetylation levels are common, we compared H3K9/14ac intensity for all active promoter and enhancer regions in two hrAPLs and two APLs. Our findings revealed increased H3K9/14ac levels at the two types of regions in hrAPLs as compared to APLs (Figure 3C), suggesting the hyperacetylation signature is not only present at PML-RARA binding sites, but even is a ubiquitous mark at other regulatory elements.

As the difference in histone H3 acetylation was most obvious at putative enhancer regions, we examined whether these regions might represent an activation signature of a unique cellular population. For this, we compared the H3K27ac occupancy landscape of hrAPLs with those of five progenitor cells from healthy individuals including normal CD34^+^ cells, promyelocytes, metamyelocytes, band neutrophils and segmented neutrophils. This revealed distinct global acetylation levels at the hrAPL enhancer sites in different progenitor population (Figure 3D), but no similar enrichments as observed in hrAPLs, suggesting that the hyperacetylation signature present in hrAPLs is not reminiscent of a specific progenitor population.

To determine enriched consensus sequences at all active enhancers with the hyperacetylation signature in hrAPLs (Figure 3C), we conducted motif screening. This discerned significant enrichment for RUNX1 and ETS consensus motifs (Figure 3E), implying transcription factors belonging to these families might assist in epigenetic marking of these regions.

Finally, we asked whether the hyperacetylation state at identified enhancers is specific for hrAPLs. For this, we compared the hrAPL H3K27ac pattern with levels of 27 AMLs with varying prognosis and cytogenetic background [25]. In order to get unbiased and precise results, we included 4 additional high-risk APLs (pt#A to D; number of leukocytes >10,000/µL) and 1 low-risk APL (pt#E). Indeed, acetylation levels at the set of hrAPL enhancers are increased as compared to acetylation at these regions in almost any other subtype of AML (*p* < 0.01 for pairwise comparisons) (Figure 3F), implying that the acetylation signature might be used to identify this particular subclass of APLs.

To allow clinical discrimination of this subset using assays that are sequencing-independent, we defined the most differential acetylated regions. Using an FDR <0.01 and *p*-value < 0.0002, we identified 22 regions specifically acetylated in hrAPLs that could potentially be used for clinical subtype stratification (Supplementary Table 1), although these results will need further confirmation in a larger cohort of hrAPLs.

Altogether these results reveal that the histone acetylation landscape of hrAPLs is different as compared to low/intermediate-risk APLs and myeloid progenitors, and generally represents a hyperacetylated histone signature.

### Deregulation in repressive histone methylation impacts on APLs with low overall survival

**Figure 4:**
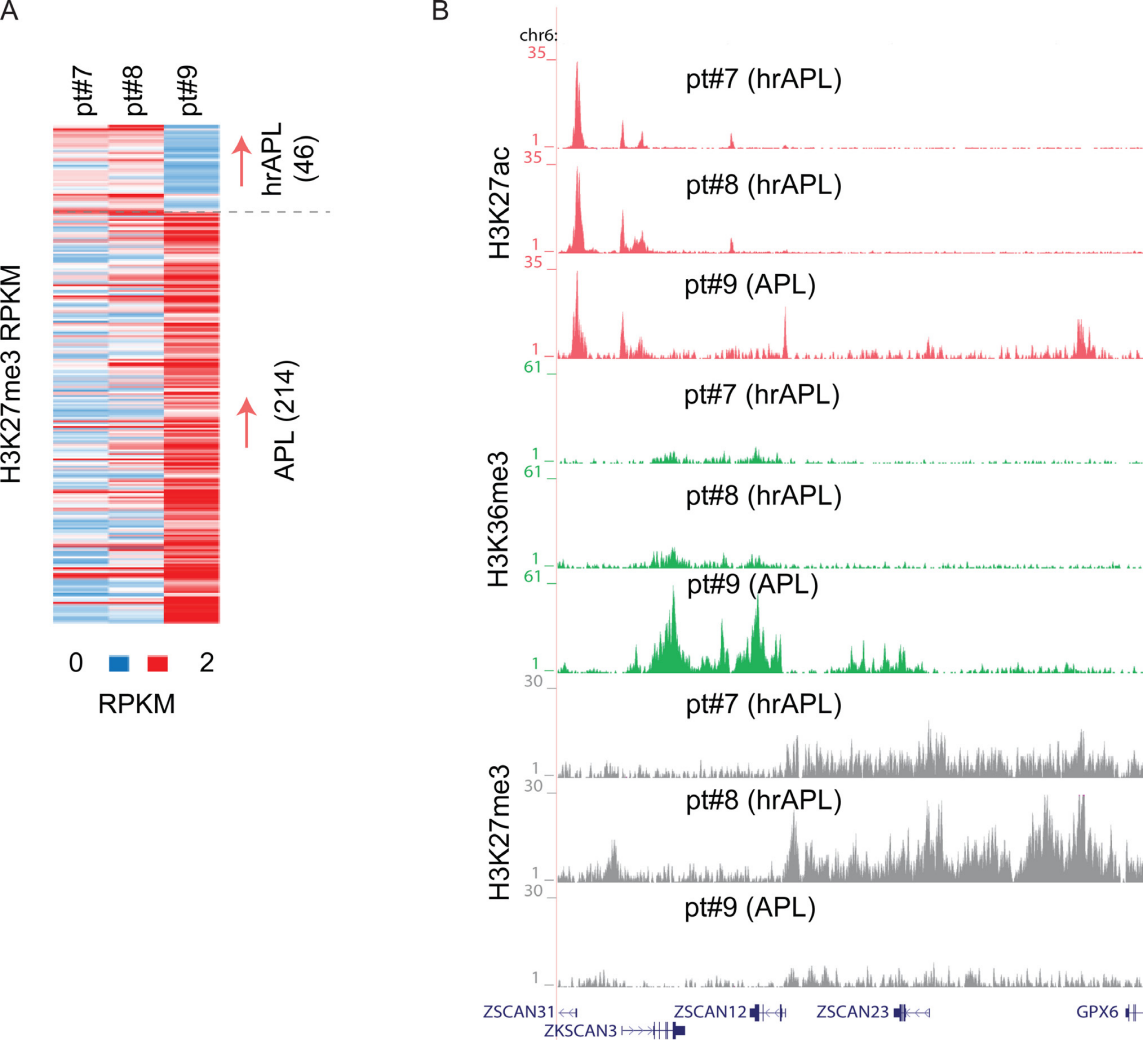
H3K27me3 alterations in high-risk APLs (hrAPLs) (**A**) Heatmap of H3K27me3 levels on differential regions between primary APL (pt#9) and hrAPL (pt#7 and pt#8) samples. (**B**) Overview of H3K27ac, H3K36me3 and H3K27me3 levels at the *ZSCAN12* genomic region in one primary APL and two hrAPL samples.

APL leukemogenesis has also been linked to H3K27me3 alterations [3, 13], suggesting an important role for those genomic regions occupied by the PRC2 complex and H3K27me3 in APL. We speculated that in hrAPLs, the hyperacetylated signature might affect the presence of repressive marks, especially H3K27me3 given the mutual exclusivity of acetylation and methylation on the same lysine. Inspecting H3K27me3 patterns in hrAPLs and APL revealed differential occupancy at a number of loci, for example, H3K27me3 hypermethylation at the *ZSCAN12*/*GPX6* genomic region (Figure 4A, 4B), but mainly hypomethylation at other (*n* = 214) differential H3K27me3 regions in hrAPLs. These results indicate that besides H3 acetylation also other epigenetic features such as H3K27me3 are altered in hrAPL and likely affect the transcriptional program.

### High-risk APLs harbor an altered DNA methylation signature

**Figure 5:**
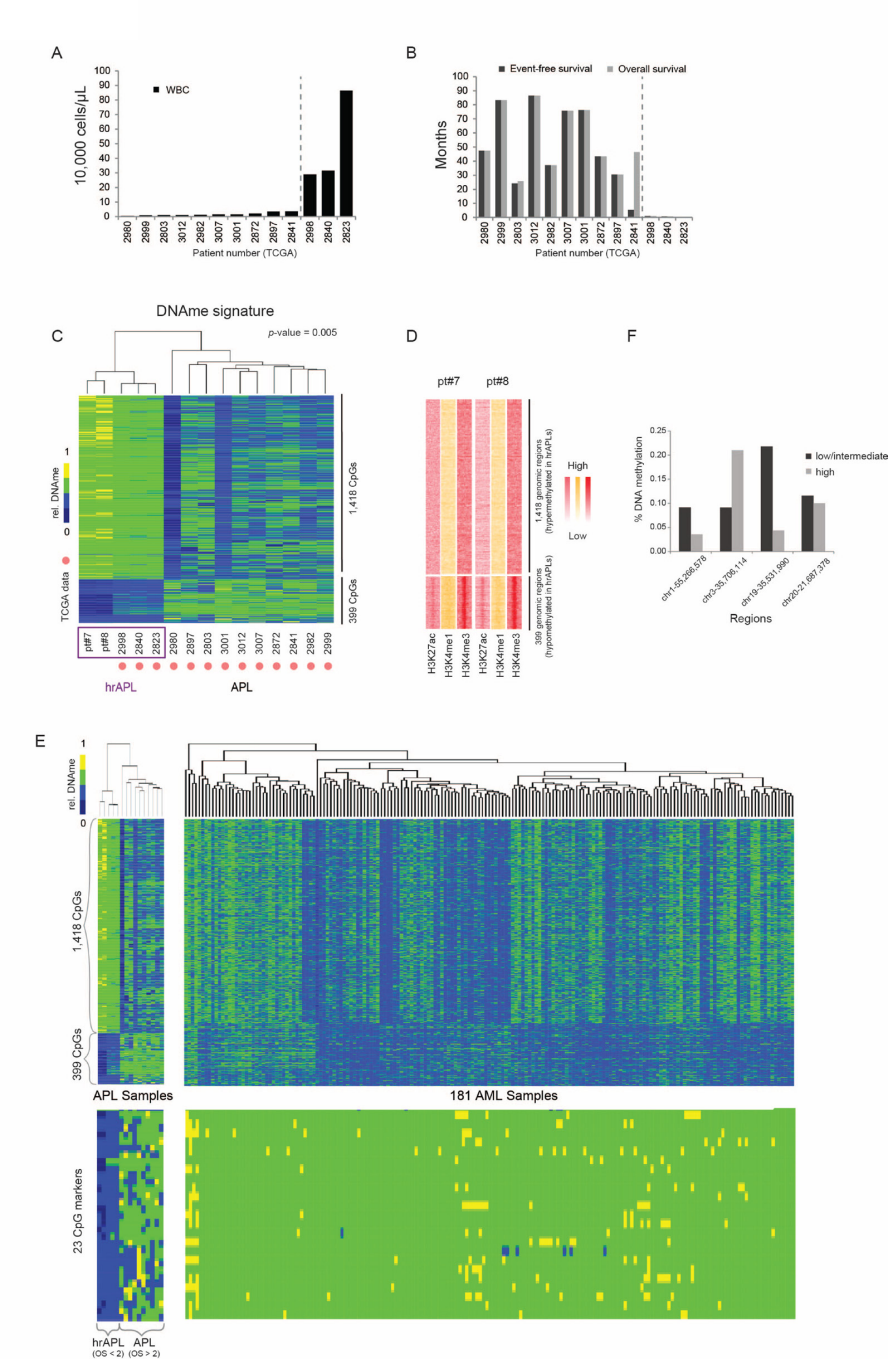
DNA methylation characteristics in high-risk APLs (hrAPLs) (**A**) White blood cell counts in 13 APL cases described and analyzed in Ley *et al.*, 2012. [26] (**B**) Event Free Survival (EFS) and Overall Survival (OS) in 13 APL cases described and analyzed in Ley *et al.*, 2012. [26] (**C**) Supervised clustering of DNA methylation patterns using 5 hrAPLs and 10 APLs. (**D**) Heatmap of histone modification density at the two clusters defined in (C). (**E**) (Top) DNA methylation patterns of 181 AMLs over the two APL clusters defined in Figure (C). (Bottom) DNA methylation patterns using 181 AML and 15 APLs over 23 CpGs hypomethylated in hrAPLs. (**F**) DNA methylation level of high-risk and low/intermediate-risk APLs [27] at 4 regions overlapping with the 23 CpG DNAme signature identified in this study.

Next, we assessed whether hrAPL and APLs presented differential global CpG methylation patterns. To extend our dataset, we included DNA methylation data of a cohort of 13 APL patients (450 K, from TCGA; [26]), of which 3 had an exceptionally high white blood cell count (>10,000/µL) and survival below 1 month that could be integrated as hrAPL cases (Figure 5A, 5B).

Supervised clustering to identify differential CpG methylation sites between hrAPLs and APLs revealed a set of 1,817 CpGs that could classify APLs into the high- or low/intermediate-risk category (Figure 5C). These included 399 hypomethylated CpG regions (CpG coverage >4 and *p*-value < 0.005) which displayed enriched H3K4me3 and H3K27ac levels in hrAPLs (Figure 5D), and 1,418 hypermethylated CpG regions. To further validate this finding, we integrated all DNA methylation data from TCGA AML samples [26]. This indicated that the unique hyper/hypo methylation signature discovered for hrAPLs is specific (Figure 5E, top). Importantly, this signature could be further filtered to 23 CpGs (CpG coverage > 4, *p*-value < 0.0007) (Supplementary Table 2; Figure 5E, bottom), which could distinguish hrAPLs from all APLs/AMLs.

To validate this signature, we examined methylation levels reported for high-, intermediate- and low-risk APLs in a separate study [27]. This showed that 3 out of 4 regions with sufficient coverage were hypomethylated in high-risk cases as compared to low/intermediate-risk APLs (Figure 5F). These results suggest that the 23 CpG hrAPL signature potentially enables PCR-based approaches to identify the hrAPL subtype at diagnosis. As for the identified Histone H3 acetylation signature above, further confirmation in a larger cohort of hrAPLs will be needed.

### MC2884 induces apoptosis in hrAPLs and remodels the epigenome

**Figure 6:**
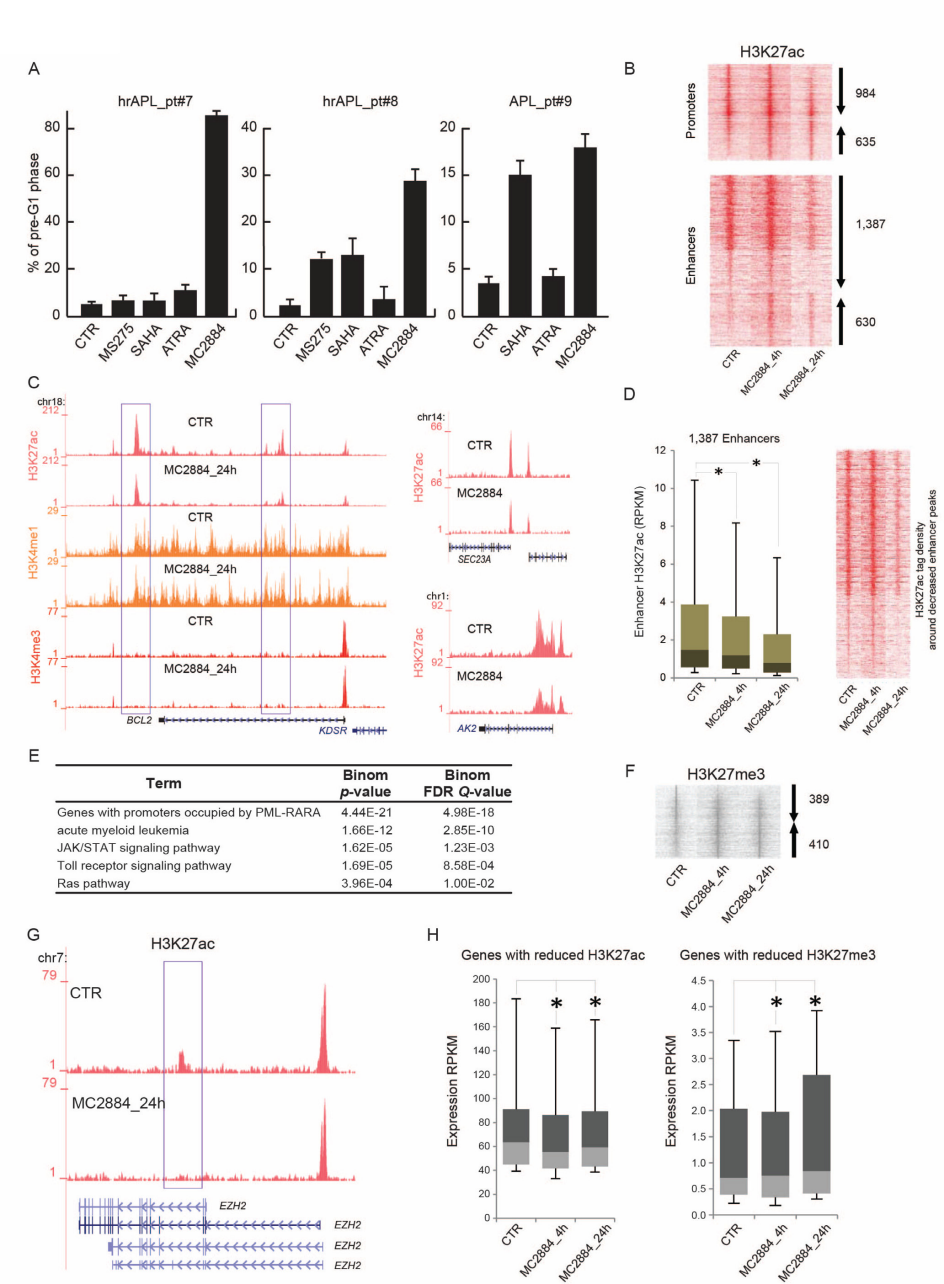
MC2884 induces apoptosis in hrAPLs and alters the epigenome (**A**) Analysis of cell death (% of cells in pre-G1 phase) induced by MC2884, HDACi (MS275 and/or SAHA) and ATRA in *ex vivo* APL blasts (24 h). (**B)** Heatmap showing H3K27ac changes at promoter and enhancer regions upon *ex vivo* MC2884 treatment of hrAPL (pt#8) cells. (**C**) Overview of epigenetic changes at the *BCL2*, *AK2* and *SEC23A* genomic regions in control or MC2884 *ex vivo* treated hrAPL cells. (**D)** (Left) Boxplot and (Right) heatmap showing H3K27ac changes at enhancer regions that show reduced occupancy levels after *ex vivo* MC2884 treatment of a hrAPL sample. ^*^ indicates significance at *p* < 0.01. (**E**) Table showing functional annotation of genes associated with enhancers that have reduced H3K27ac after *ex vivo* MC2884 treatment of hrAPL (pt#8). (**F**) Heatmap showing H3K27me3 changes at genomic regions upon *ex vivo* MC2884 treatment of hrAPL cells. (**G**) Overview of H3K27ac at the *EZH2* gene in control or MC2884 *ex vivo* treated hrAPL cells; (**H)** Expression of genes associated with enhancers (distance to gene <50 kb and RPKM sum >100; left) reduced in H3K27ac or of genes associated with promoters with reduced H3K27me3 (RPKM sum > 0.5; right). ^*^ indicates significance at *p* < 0.01.

Since hrAPLs display multiple epigenome deregulations, we wondered whether a multi epi-drug approach targeting HATs and PRC2 would be beneficial. We identified MC2884, a novel HAT/EZH2 inhibitor (Petraglia *et al.*, in press), as a promising candidate. We evaluated MC2884-induced apoptosis in the hrAPLs (pt#7 and pt#8) as well as in an ATRA-sensitive APL (pt#9) after 24 hours *ex vivo* treatment. Our results show that the hrAPLs did not undergo efficient apoptosis (as measured by the % of cells in the pre-G1 phase) *ex vivo* in response to HDACi’s such as SAHA or MS275 [28], when compared to ATRA-sensitive blasts (pt#9). In contrast, *ex vivo* MC2884 treatment in hrAPLs was accompanied by remarkable apoptosis induction (Figure 6A).

As the hrAPLs harbor an aberrant H3K27ac, H3K9/14ac and H3K27me3 signature, we inquired whether MC2884 would reset acetylation and H3K27me3 patterns and thereby trigger the onset of apoptosis. We therefore treated pt#8 blasts with MC2884 *ex vivo* for 4 and 24 h, and examined H3K27ac and H3K27me3 levels. We identified 3,636 H3K27ac regions (Figure 6B) for which occupancy was changed (FC > 3×STDEV, % gain, % loss), such as at the *BCL2*, *SEC23* and *AK2* genomic regions (Figure 6C). Amongst the changed regions, 1,387 represented enhancer regions decreased in H3K27ac (Figure 6D). Functional annotation of these decreased enhancer regions revealed enrichment for genes associated with AML, PML-RARA binding, JAK/STAT signaling, Toll-like receptor signaling, and the RAS pathway (Figure 6E).

In addition to H3K27ac changes, we observed 799 H3K27me3 regions for which occupancy was changed (FC > 3×STDEV, % gain, % loss) (Figure 6F), suggesting that MC2884 treatment might alter the function of histone H3 lysine 27 both by reducing acetylation at this residue as well as methylation. Interestingly, reduced H3K27ac was also observed at a putative enhancer region of *EZH2* (Figure 6G), suggesting that MC2884-chromatin action also affects *EZH2* gene transcription and further changes polycomb complex localization. Indeed, expression of *EZH2* was reduced, similar as for other genes associated with reduced enhancer acetylation (Figure 6H, left). In contrast, genes associated with reduction in H3K27me3 increased in expression (Figure 6H, right). Together, these results reveal that MC2884 treatment reshapes the epigenome of hrAPLs and increases apoptosis.

## DISCUSSION

Treatment of APL has significantly advanced over the last decade due to increased knowledge on the molecular mechanisms underlying APL pathogenesis and the widespread application of ATRA and arsenic trioxide (ATO) in treatment protocols. While low/intermediate-risk APL patients have benefited from these developments, especially the reduced usage of chemotherapy, clinical therapy for high-risk APL patients often still include cytotoxic chemotherapy [20]. Here, by performing an in-depth transcriptomic and epigenomic analysis, we aimed to point out major causes of severe drug resistance in these high-risk APLs (hrAPLs) and identify entry points for the application of epi-drugs.

As no common genetic signature could be identified by our genomic scan, we focused on the epigenomic comparison between high-risk and low/intermediate-risk APL samples. Our findings revealed hrAPL-specific alterations in histone acetylation, H3K27me3 and DNA methylation. Given these specific epigenomic features, we tried to establish hrAPL-specific epigenetic signatures that could potentially be used as biomarker. For histone acetylation, we identified 22 hyperacetylated regions that seemed to be hrAPL-specific, and for DNA methylation we proposed a signature of 23 CpGs that distinguishes hrAPLs from other APLs/AMLs. Additional samples and confirmation in different cohorts will be needed to support these regions as real biomarkers. Nevertheless, it suggests that histone acetylation level has similar potential to classify samples as DNA methylation signatures.

The prominent signature of hyperacetylation of histones at a set of defined promoters and enhancers in high-risk APLs suggests a more open chromatin structure in hrAPLs. The hyperacetylation signature was also observed at PML-RARA binding sites, which seemed counterintuitive given the previously reported recruitment of histone deacetylases by us and others [8, 9, 13, 29]. Moreover, it is surprising that despite a hyperacetylation signature at PML-RARA binding sites, histone acetylation is still increased upon ATRA treatment. Previously, we have shown that for another AML associated fusion protein (AML1-ETO) both acetyltransferase and deacetylase enzymes are present at its binding site [30]. This could well be the case in this situation as we already previously reported the acetyltransferase p300 might be present at PML-RARA binding sites [31] to mediate histone acetylation. Maybe through stabilization of its binding or increased activity, hyperacetylation at the PML-RARA binding regions is established in hrAPLs. However, it can still be expected that also HDACs recruited by PML-RARA will be present. These will be removed upon ATRA treatment and subsequent PML-RARA degradation, resulting in an even further increase in acetylation.

Given the hyperacetylation signature and the absence of a clear ATRA response (which would even further increase acetylation), inhibition of HDAC activity which has been previously proposed to treat APL [9, 32–36] would likely be ineffective. As we also observed other epigenetic alterations in hrAPLs as compared to APLs, especially for H3K27me3, our analysis suggested that a combination of epigenetic drugs that affect histone acetyltransferase and H3K27 methyltransferase activity might be beneficial. Using the novel HAT/EZH2 inhibitor MC2884 confirmed that altering these hrAPL-specific epigenomic features could indeed inhibit cell growth. These results suggest that epigenome-based patient stratification, in this case hrAPLs may provide a novel strategy to be coupled with genome analyses to “personalize” precision medicine.

## METHODS

### Next generation sequencing (NGS) experiments

Samples for NGS experiments were obtained from different laboratories but have been processed similarly, with standard operating procedures set by BLUEPRINT at the start of the project using extensively validated and documented antibody batches and other reagents. Moreover, all samples underwent strict quality control at different levels (e.g. sequence quality, read depth, visual inspection) before they were included in the downstream analysis pipeline. The exact procedures and quality filters can be found at the BLUEPRINT website (www.blueprint-epigenome.eu).

### Chromatin immunoprecipitation (ChIP)

ChIP extracts preparation and procedures have been carried out following IHEC procedures using Diagenode antibodies and as reported [13, 29]. The fold enrichment of H3K27ac and H3K27me3 ChIP’ed DNA was evaluated. Primer sequences were as follows: BCL2 promoter region (at -410 and -282 from +1), forward (5′- GTG TTC CGC GTG ATT GAA GAC-3′) and reverse (5′- CAG AGA AAG AAG AGG AGT TAT AA-3′); for chr21:44,496,353-44,496,918/CBS region, forward (5′- CGC AGA ACA GTC GCC TTG-3′) and reverse (5′- GTC CAG AGC ACG ATG TTT GG-3′); for chr17:4,938,577-4,939,058/SLC52A1 region, forward (5′- CGA GTT GGA GAG GGG AGT G-3′) and reverse (5′- AAC AAA ACC CCA GCT GTG TG-3′).

### RNA extraction and RT-PCR

Total RNA was extracted with Trizol (Invitrogen) and converted into cDNA using VILO (Invitrogen). For amplification, the following primers were used: EZH2, forward (5′-CATCATAGCTCCAGCTCCCG-3′) and reverse (5′-CATCCCGGAAAGCGGTTTTG-3′); EED, forward (5′-CGATTTGCGACAGTGGG-3′) and reverse (5′-CAGGTGCATTTGGCGTG-3′); SUZ12, forward (5′-GTCCTGCTTGTGAAAGTTTGC-3′) and reverse (5′-CAAATGTCTTTTCCCCATCCT-3′); BCL2, forward (5′-GAACTGGGGGAGGATTGTGG-3′) and reverse (5′-CAGCCTCCGTTATCCTGGAT-3′); GAPDH, forward (5′-TCAACGGGAAGCCCATCACCA-3′) and reverse (5′-ACGGAAGGCCATGCCAGTGA-3′).

### Whole-genome bisulfite sequencing

Genomic DNA (1–2 μg) was spiked with unmethylated λ DNA (5 ng of λ DNA per μg of genomic DNA) (Promega). The DNA was sheared by sonication to 50–500 bp using a Covaris E220 and fragments of size 150–300 bp were selected using AMPure XP beads (Agencourt Bioscience Corp.). Genomic DNA libraries were constructed using the Illumina TruSeq Sample Preparation kit (Illumina Inc.) following the Illumina standard protocol: end repair was performed on the DNA fragments, an adenine was added to the 3′ extremities of the fragments and Illumina TruSeq adapters were ligated at each extremity. After adaptor ligation, the DNA was treated with sodium bisulfite using the EpiTexy Bisulfite kit (Qiagen) following the manufacturer’s instructions for formalin-fixed and paraffin-embedded (FFPE) tissue samples. Two rounds of bisulfite conversion were performed to assure a high conversion rate. An enrichment for adaptor-ligated DNA was carried out through 7 PCR cycles using the PfuTurboCx Hotstart DNA polymerase (Stratagene). Library quality was monitored using the Agilent 2100 BioAnalyzer (Agilent), and the concentration of viable sequencing fragments (molecules carrying adaptors at both extremities) estimated using quantitative PCR with the library quantification kit from KAPA Biosystem. Paired-end DNA sequencing (2 × 100 nucleotides) was then performed using the Illumina Hi-Seq 2000.

The data are publicly available and can be accessed via the Blueprint DCC: http://dcc.blueprint-epigenome.eu/#/experiments.

### Peak calling and identification of promoter and enhancer regions

For peak calling the BAM files were first filtered to remove the reads with mapping quality less than 15, followed by fragment size modeling (http://code.google.com/p/phantompeakqual-tools/). The peak-calling algorithm MACS2 (http://github.com/taoliu/MACS/) was used to detect the binding sites for the three studied histone marks at default *q*-value (5.00e-02). H3K4me1 peaks were called using the broad setting of MACS2 while peaks for marks H3K27ac, H3K4me3 and transcription factor PML-RARA were called using the default (narrow) setting.

In order to identify promoter and enhancer regions, non-overlapping H3K4me3 and H3K4me1 bound regions were taken into account, respectively. The common promoter and enhancer regions across the hrAPL samples were computed using the in-house script intersectbed. pl. Further, normalized tag enrichment for H3K27ac and H3K27me3 was used to mark active and inactive promoter and enhancer regions. Also, normalized tag enrichment for H3K27ac and H3K9/14ac in common enhancer regions was used to compute differentially active regions across different samples (pt#7, pt#8 and pt#9) and cell types (CD34^+^, promyelocytes, metamyelocytes, band neutrophils and segmented neutrophils). The intensity graphs were generated using the in-house script makeColorprofiles.pl. Discriminating hyperacetylated regions were identified using DESeq package (http://bioconductor.org/packages/release/bioc/html/DESeq.html) and selection for regions with an FDR < 0.01 and *p*-value < 0.0002 between hrAPL and all other samples (APL and AML). Motif analysis was performed as before [30].

The data are publicly available and can be accessed via the Blueprint DCC: http://dcc.blueprint-epigenome.eu/#/experiments.

### RNA-seq alignment and expression analysis

RNA-seq reads were aligned using GSNAP [37] using non-default parameters -m 1 - N 1 -n 1 -Q -s Ensembl_splice_68. RNA-seq library data was initially subjected to a quality control step, where, based on read distribution over the annotated genome, libraries that are outliers were identified and discarded from further analysis. For expression analyses, reads were aligned to the Ensembl v70 (GRCh37.70) human transcriptome using Bowtie. Quantification of gene expression was performed using MMSEQ [38].

The data are publicly available and can be accessed via the Blueprint DCC: http://dcc.blueprint-epigenome.eu/#/experiments.

### SNP calling and identification of potential functional SNPs

SNP calls were identified using the CNAG in-house pipeline based on WGBS data. The list of variant calls was reduced by applying a number of filters, starting from the number of tags (≥20) and Phred score (≥40) on the SNP calls from WGBS. The reduced list was then given as an input to the CHASM web server (http://www.cravat.us/ version 3.1) for functional annotation and only the missense variants present in driver genes (as identified by Vogelstein) [39] at MAF ≤ 5% (1000 G) were selected. Further, only the variants reported in ChIP-seq and RNA-seq tracks by applying computational tools samtools (*mpileup* option) and GATK along with the variations confirmed through visual inspection of the tracks using IGV were selected.

The data are publicly available and can be accessed via the Blueprint DCC: http://dcc.blueprint-epigenome.eu/#/experiments.

### Identification of signature methylation regions

Cytosine methylation level was called for every cytosine site in different sequence contexts such as CHH, CXG and CG (H denotes A, T, or C and X denotes A or T). Due to the fact that symmetric methylation level is the common case for CG methylation, we focused on CG methylation and excluded non-CpG methylation for further analysis. Within a symmetric CG context, CG pairs on both forward and reverse strands were combined and a CpG coverage threshold of ≥5 was used. Further, the methylation samples pt#7 and pt#8 were grouped together with the 3 APL samples of low OS from TCGA [26] and compared with the APL samples. We selected the regions for which CG methylation was determined across all samples and applied the Wilcoxon test to determine statistically significant different CpGs between the two groups. A cut-off of the *p*-value of 0.005 was applied as the selection criteria and regions were plotted. To identify the most discriminating CpGs, we applied the same test at a more stringent cut-off *p*-value of 0.0007. Moreover, only the CpG regions that had beta values in all 181 AML samples were selected.

The metadata of the APL and AML samples can be found in Supplementary Table 3.

### Cell cycle analysis

For cell cycle analyses, cells were plated (2 × 10^5^ cells/mL) and after stimulation (performed as indicated in the text) were harvested, centrifuged at 1,200 rpm for 5′ and resuspended in 500 μL of a hypotonic solution containing 1X PBS, Sodium Citrate 0.1%, 0.1% NP-40, RNAase A and 50 mg/mL Propidium Iodide (PI). After 30’ at room temperature (RT) in the dark, samples were acquired by FACS-Calibur (BD Biosciences, San Jose, CA, USA) using CellQuest software (BD Biosciences). The percentage in different phases of the cell cycle (and pre-G1 as a measure of apoptosis) was determined by ModFit LT V3 software (Verity). All experiments were performed in triplicate.

## SUPPLEMENTARY MATERIALS TABLES




