# Supplementary material for: Multi-omics profiling reveals a distinctive epigenome signature for high-risk acute promyelocytic leukemia
Source: Oncotarget. 2018 May 22;9(39):25647–60. doi: 10.18632/oncotarget.25429 (PMC5986653; doi:10.18632/oncotarget.25429)
Supplement: Supplementary file 1 [file oncotarget-09-25647-s001.pdf]

Multi-omics profiling reveals a distinctive epigenome signature for high-risk acute promyelocytic leukemia

SUPPLEMENTARY MATERIALS

Supplementary Table 1: Hyperacetylated regions (hg19) in hrAPLs

chromosome	start	end
chr1	95181929	95184074
chr1	109626120	109627905
chr1	249221773	249222010
chr11	57158202	57159391
chr14	61416231	61417467
chr14	105391316	105391725
chr17	70982083	70984392
chr17	71061705	71070236
chr2	27060445	27061863
chr2	102056668	102060845
chr2	234072888	234075888
chr20	2665743	2666563
chr22	50153595	50155770
chr3	41011746	41014498
chr6	10087785	10088550
chr6	82468660	82469114
chr6	139948334	139949640
chr6	140000376	140002294
chr7	29323864	29325989
chr9	94907894	94910771
chrX	37605417	37607058
chrX	151152531	151154618

Supplementary Table 2: Hypomethylated CpGs (hg19) in hrAPLs

chromosome	start	end
chr1	1052949	1052950
chr1	12655992	12655993
chr1	17287501	17287502
chr1	55266578	55266579
chr2	237477262	237477263
chr3	35706114	35706115
chr3	57198244	57198245
chr4	3516534	3516535
chr4	163085348	163085349
chr6	73972852	73972853
chr12	57630107	57630108
chr12	114841708	114841709
chr14	24779959	24779960
chr16	1429015	1429016
chr16	20817501	20817502
chr16	46919112	46919113
chr16	67197640	67197641
chr17	39845949	39845950
chr19	3275394	3275395
chr19	35531990	35531991
chr19	39056084	39056085
chr20	21687378	21687379
chr20	62084626	62084627

Supplementary Table 3: BLUEPRINT samples used in Singh *et al.* See Supplementary_Table_3
